# Supplementary material for: Influence of head morphology and natural postures on sound localization cues in crocodilians
Source: R Soc Open Sci. 2019 Jul 10;6(7):190423. doi: 10.1098/rsos.190423 (PMC6689610; doi:10.1098/rsos.190423)
Supplement: Biometry and dimensions of the animals and skulls used in the present study. [file rsos190423supp1.pdf]

rsos.royalsocietypublishing.org Proc R Soc B 000000

Influence of head morphology and natural postures on sound localization cues in crocodilians

L. Papet[1,2], N. Grimault[1], N. Boyer[2], N. Mathevon[2]
[1]Centre de Recherche en Neurosciences de Lyon - Équipe Cognition Auditive et Psychoacoustique, CNRS UMR 5292, Lyon, France.
[2]Equipe Neuro-Ethologie Sensorielle ENES / NeuroPSI, CNRS UMR 9197 - University of Lyon / Saint-Etienne - Saint-Etienne, France.

February 2019

Electronic Supplementary Material 1

Table 1. Biometry and dimensions of the animals and skulls used in the present study.

**Material**	**Species**	**Total length (cm)**	**Rostral snout length (cm)**	**Rostral snout width (cm)**	**Interaural distance (cm)**	**Weight (kg)**
Juvenile crocodile	*Crocodylus niloticus*	91.5	6.9	5.1	4.7	2.7
Juvenile caiman	*Caiman latirostris*	68.5	4.4	5.0	3.9	1.5
Adult skull	*Crocodylus niloticus*	×	22.7	10.4	6.0	×
Young skull	*Crocodylus niloticus*	×	16.5	6.5	4.5	×
Juvenile skull	*Crocodylus niloticus*	×	6.9	3.8	2.4	×
